# Omnidirectional Underwater Camera Design and Calibration

**DOI:** 10.3390/s150306033

**Published:** 2015-03-12

**Authors:** Josep Bosch, Nuno Gracias, Pere Ridao, David Ribas

**Affiliations:** Computer Vision and Robotics Group, Centre d'Investigació en Robòtica Submarina, Parc Científic i Tecnològic, Universitat de Girona, 17003 Girona, Spain; E-Mails: ngracias@eia.udg.edu (N.G.); pere@eia.udg.edu (P.R.); dribas@udg.edu (D.R.)

**Keywords:** omnidirectional, underwater, camera, calibration, housing, OMS, image stitching, image blending, panorama construction

## Abstract

This paper presents the development of an underwater omnidirectional multi-camera system (OMS) based on a commercially available six-camera system, originally designed for land applications. A full calibration method is presented for the estimation of both the intrinsic and extrinsic parameters, which is able to cope with wide-angle lenses and non-overlapping cameras simultaneously. This method is valid for any OMS in both land or water applications. For underwater use, a customized housing is required, which often leads to strong image distortion due to refraction among the different media. This phenomena makes the basic pinhole camera model invalid for underwater cameras, especially when using wide-angle lenses, and requires the explicit modeling of the individual optical rays. To address this problem, a ray tracing approach has been adopted to create a field-of-view (FOV) simulator for underwater cameras. The simulator allows for the testing of different housing geometries and optics for the cameras to ensure a complete hemisphere coverage in underwater operation. This paper describes the design and testing of a compact custom housing for a commercial off-the-shelf OMS camera (Ladybug 3) and presents the first results of its use. A proposed three-stage calibration process allows for the estimation of all of the relevant camera parameters. Experimental results are presented, which illustrate the performance of the calibration method and validate the approach.

## 1. Introduction

In the last few years, omnidirectional cameras have received increasing interest from the computer vision community in tasks such as augmented reality, visual surveillance, motion estimation and simultaneous localization and mapping (SLAM). The wide field of view (FOV) provides a comprehensive view of a scene. However, the use of these cameras underwater is still at a very early technological stage.

The use of omnidirectional cameras in underwater environments opens the door to several new technological applications in fields as diverse as underwater robotics, marine science, oil and gas industries, underwater archeology and science outreach. As an example, underwater panoramic images can be used to create virtual reality tours of zones of special interest, like shipwrecks or underwater nature reserves. In the first case, it would be an attractive and innovative tool to bring archeology closer to the general public, and in the latter, it can be an enticing way to promote awareness for the preservation of a specific region.

**Figure 1.**
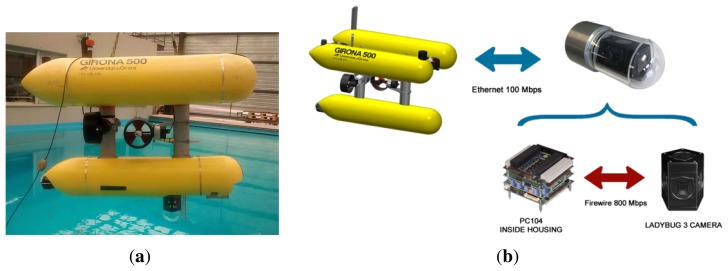
Integration of the omnidirectional camera in the Girona500 AUV. (**a**) The omnidirectional camera integrated with the Girona500 AUV in the CIRS (Underwater Robotics Research Centre) water tank; (**b**) scheme of the communications between the Girona500 AUV and the omnidirectional camera.

For underwater robotics, omnidirectional cameras are expected to have a large impact in both remotely-operated vehicles (ROVs) and autonomous underwater vehicles (AUVs) [1]. It will allow ROVs to be piloted directly through the images captured by the omnidirectional cameras through virtual reality headsets. This immersive experience will extend the pilots spatial awareness and reduce the usual orientation problems during missions. For AUVs, the wide FOV of the cameras is very convenient for visual SLAM and mapping tasks, especially in confined or cluttered environments. In particular, the omnidirectional camera presented in this paper has been developed in the framework of the MORPH (Marine robotic systems of self-organizing, logically linked physical nodes) EU-FP7 project [2]. One of the tasks of the project involves cooperative navigation between AUVs. For this purpose, the camera will be integrated into the Girona500 AUV (Figure 1a) [3], enabling the tracking and pose estimation of other robots navigating close to it.

This paper presents an underwater omnidirectional multi-camera system (OMS) based on a Point Grey's Ladybug 3 [4] camera. The Ladybug 3 comprises six individual cameras and is designed for land applications. In order to be used underwater, structural changes are required in the camera itself, as well as the manufacturing of a custom waterproof housing. As a consequence, the factory-provided calibration is not valid, and a new custom calibration procedure for underwater OMS needs to be developed.

Calibration is a mandatory step for most camera applications. It enables the use of many computer vision tools for tasks, such as 3D reconstruction or motion estimation. Calibration for multi-camera systems typically covers two different sets of parameters: intrinsic parameters, concerning the image formation geometry for each individual camera, and extrinsic parameters, which describe the relative positions and orientations between cameras. In omnidirectional multi-camera systems, the calibration of the extrinsic parameters is an important challenge, due to the usual small overlap between neighboring cameras.

In this paper, the complete calibration is done in three different stages. The first consists of the estimation of the intrinsic parameters, which is done separately for each single camera in air and without the waterproof housing. The second stage consists of the estimation of the extrinsic parameters, also done in the same conditions as the first step. Finally, the last stage takes place underwater and estimates the camera pose with respect to the waterproof housing.

This calibration procedure is done in three stages rather than in a single combined step. The reason behind this is two-fold. Firstly, it allows for a smaller number of parameters to be estimated in each individual step, thus avoiding unwanted correlations among the parameter estimates. Secondly, it allows the use of image sets captured in air, for the estimation of the parameters that are not related with the underwater housing. This way, the intrinsic and extrinsic parameters are not affected by disturbances, such as the non-modeled geometric inaccuracies of the waterproof housing. Furthermore, it is significantly easier to find feature points in images captured in air than in water, due to light absorption and varying illumination conditions. The use of a larger number of well-spread feature points contributes to a higher calibration accuracy.

Different target applications of the OMS impose different requirements on the calibration accuracy. When used for science outreach and visualization purposes, the effort will be put in creating panoramas with the minimal amount of distracting visual artifacts. By contrast, when the OMS is used for object recognition or tracking, the effort will concentrate on achieving the best model possible for the image formation geometry.

### 1.1. Related Work

Camera calibration has been a topic of research since cameras have started being used for metrology [5,6]. There is a vast body of literature in the photogrammetry field [7–9] that focuses on modeling all of the relevant aspects of the image formation, towards obtaining high accuracy models. However, such a vast literature is almost completely devoted to aerial and land applications. Comparably fewer references can be found for underwater metrology, especially for the case of non-conventional camera systems, like OMS. Of particular relevance to this paper is the existing work on the modeling of wide-angle and fish-eye lenses. Kannala and Brandt [10] presented a generic geometric model and a calibration method based on a planar calibration pattern suitable for both fish-eye lenses and conventional cameras. Scaramuzza *et al.* [11] propose another calibration method using a planar grid assuming that the image projection function can be described by a Tailor series expansion. Mei and Rives [12] propose a model based on the exact theoretical projection function and with the addition of parameters to model real-world errors.

Most omnidirectional cameras can be divided into two main groups [13]: central omnidirectional cameras, which strictly satisfy the single-viewpoint property, and non-central omnidirectional cameras. The first group is formed by all catadioptric systems, combinations of wide camera lenses and parabolic or hyperbolic mirrors. The later group, known as omnidirectional multi-camera systems (OMS) or polycameras, is formed by cameras composed of a cluster of individual cameras pointing to different directions in order to cover the maximum FOV possible. The first group of omnidirectional cameras is usually less expensive than an OMS, but their resolution tends to be lower and nonuniform. Typically, the resolution of catadioptric cameras is maximum in the center of the image and decreases significantly when approaching the corners. Furthermore, they are not as compact as an OMS, and its encapsulation to be used underwater is not trivial.

A few authors have analyzed the calibration of OMS with minimal overlapping between cameras. Kumar *et al.* [14] propose a calibration methodology for non-overlapping cameras using a mirror and a standard checker board. Ikeda *et al.* [15] propose a calibration method based on a calibration pattern and the use of a laser measurement system. Li *et al.* [16] presented a MATLAB toolbox for OMS calibration. This toolbox estimates both intrinsic and extrinsic parameters of the omnidirectional camera through the use of a custom descriptor-based calibration pattern rather than a standard pattern. The authors claim that the use of the custom pattern enables many more features of varying scales to be easily detected.

Regarding underwater cameras, very few works on omnidirectional underwater cameras can be found. Yamashita [17] proposed an omnidirectional underwater stereo sensor based on individual conventional video cameras and hyperboloid mirrors inside an acrylic cylindrical waterproof case. As mentioned before, this solution has not been adopted, as the use of an OMS allows one to capture panoramas in higher resolution and is more uniformly distributed. al waterproof case. As mentioned before, this solution has not been adopted, as the use of an OMS allows one to capture panoramas in higher resolution and is more uniformly distributed.

However, many authors have worked with underwater cameras and faced similar challenges due to the image distortion caused by the changes in the refractive indexes when the rays of light go through the waterproof housing. Kunz and Singh [18] examined the challenges that pressure housing interfaces introduce, focusing on hemispherical interfaces. They propose a camera calibration in two steps: a first traditional in-air calibration and the second step of adding the terms accounting for refraction. For hemispherical interfaces, there are three degrees of freedom due to the camera position inside the housing, apart from its radius and thickness, which can be measured physically. Through an optimization procedure and with the use of any standard calibration pattern, the three degrees of freedom are easily determinable. Sedlazeck and Koch [19] used mainly the same model presented by Kunz and Singh, but applied to flat ports. Similarly to [18], only three degrees of freedom are considered, corresponding to the plane orientation, plus a parameter *d* corresponding to the distance between the camera and the interface. Both works use the same approach for the underwater housing calibration as the one used in this paper. However, the geometry of the housing developed in this paper and, therefore, its ray-tracing study are significantly more complex than the ones studied previously.

### 1.2. Contributions

The main contributions of this paper are:

1. A new calibration method applicable to multiple non-overlapping camera systems in both out of the water and underwater systems. This method has the following advantages:
  (a) It overcomes the need of the cameras to see a calibration pattern entirely to compute accurately its intrinsic parameters.
  (b) It is not required during the extrinsic parameters calibration that a calibration pattern must be seen entirely by different cameras at the same time. This allows one to calibrate cameras with non-overlapping FOV.
  (c) It can handle the distortions introduced by a waterproof housing thanks to a ray tracing study.
2. The proposal and experimental validation of a compact underwater housing that does not block or limit the full FOV of the omnidirectional camera.
3. The development of an open source Linux driver [20] and a robot operating system (ROS) [21] package [22] for a Ladybug 3 camera or similar. This kind of driver was only available for Windows OS and was under the copyright of Point Grey Research Inc.

The rest of the paper is organized as follows. Section 2 presents the design of the camera and housing. Section 3 describes the single camera calibration problem. Section 4 presents the procedure used to calibrate the extrinsic parameters of all of the cameras. Section 5 introduces the challenges of the use of an omnidirectional camera underwater. In Section 6, the results of the calibration are presented. In the last section, we draw the conclusions of this work.

## 2. Camera Design

**Figure 2.**
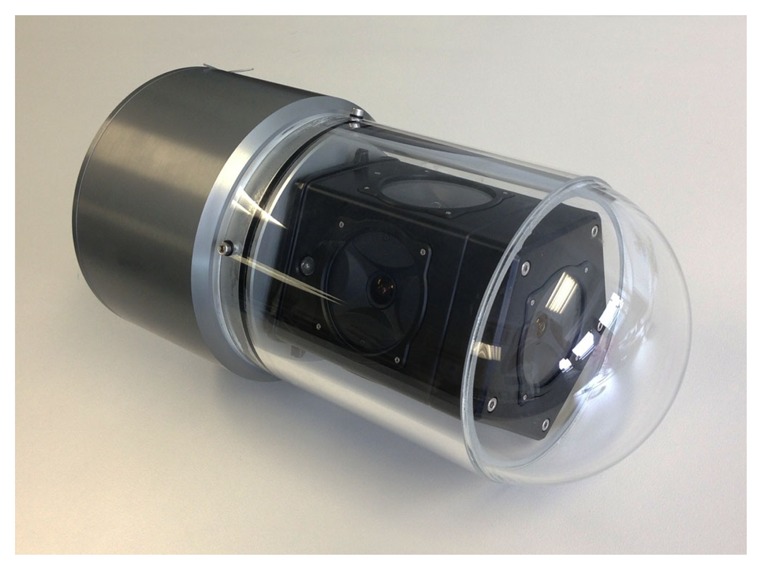
Final design of the omnidirectional underwater camera.

A custom housing has been designed for the Ladybug 3 camera to make it submersible to a water depth of 60 m. The housing is composed of a transparent poly-methyl methacrylate (PMMA) dome, which contains the camera, and a body made of aluminum alloy, which contains a small form factor computer, dedicated to processing the video feed (Figure 2).

**Figure 3.**
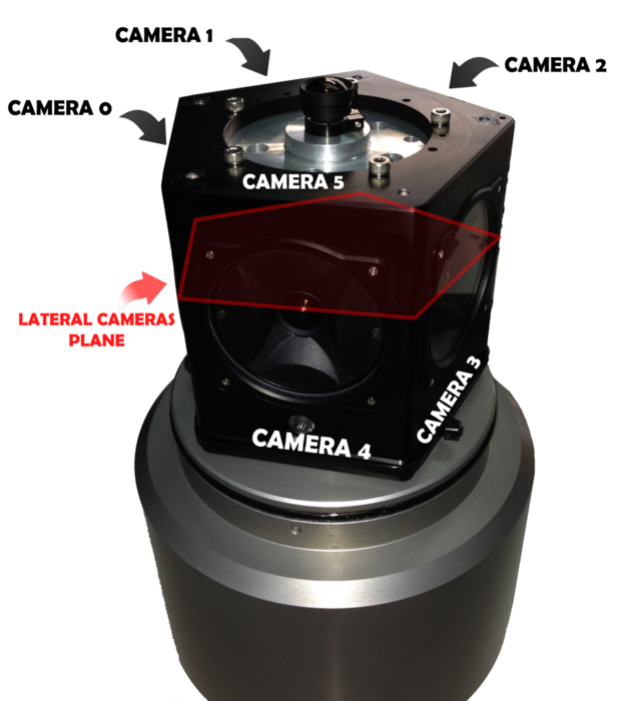
Arrangement of the cameras. The red overlay indicates the plane where the optic centers of the five lateral cameras are located (identified as Camera 0 to Camera 4). The last camera (Camera 5) has its optical axis approximately perpendicular to this plane.

The Ladybug 3 camera comprises six individual cameras (Figure 3). Five of these cameras, referred to as the lateral cameras (and numbered 0 to 4), have the optical centers on the same plane. Their optical axes also lie in the same plane with a 72° separation between neighboring cameras. The remaining camera, numbered as 5, points at the normal direction of the plane.

Each camera contains a 2 MPixel sensor, making a total of 12 MPixel for every frame captured. The images are acquired by a high-end, small form factor computer inside the housing. The role of this computer depends on the aim of the mission. When the aim of the mission is to record panoramic images of the sea, the computer will store the images without any further processing. By contrast, when the camera is used as a real-time navigation sensor, it will perform all of the image processing and send only the valuable information to the host vehicle, such as, for example, the relative position of another robot. The communication between the camera and the robot is done through an Ethernet (100 Mb/s) connection (Figure 1b).

One of the most important aspects to take into account when designing the housing is the presence of strong refraction effects on the optical rays due to the changes of media. A ray of light coming from the water changes its direction twice before reaching the sensor, as it must pass through two medium transitions (water-PMMA and PMMA-air). The change of direction is described by Snell's law [23], and it depends on two factors: the angle between the incident ray and the normal of the surface at the incidence point and the refraction indexes of the two media. Therefore, the refractions of the rays of light depend strongly on the geometry of the housing. These refractions affect the FOV of the individual cameras and the amount of overlapping between the images. Given the fact that the original camera lenses are designed for land applications, the use of an underwater housing may result in blind spots in the spherical view.

The two most typical geometries used when designing an underwater camera housing are flat and hemispherical interfaces. Flat interfaces are less expensive to manufacture and easy to mount, but they introduce important bending in the rays, which reduces the FOV of the cameras. By contrast, for an hemispherical interface with its center on the exact optical center of the camera, the incident angle and the normal of the surface are exactly the same for all of the rays, and no bending is produced during the transition. However, perfect hemispherical interfaces are difficult to produce and to mount at the exact desired position.

**Figure 4.**
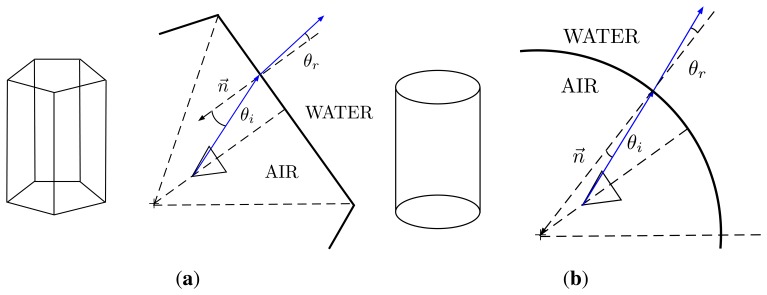
Comparison between a pentagonal prism and a cylinder as view-port options for the lateral cameras. When compared with the flat view-ports of the pentagonal prism, the cylinder has the advantage of being less affected by the refractions of the media transitions, along one of the directions. (**a**) Pentagonal prism shape; (**b**) cylindrical shape.

Due to the geometry of the Ladybug 3 camera, a transparent dome has been designed, composed of two pieces joined together with an adhesive. The first piece is a cylinder and covers the whole FOV of the five lateral cameras. A cylindrical interface has the advantage of allowing larger FOV (in one direction) for the five lateral cameras when compared with the option of having five individual flat ports (Figure 4) and is easier and less expensive to produce. The cylindrical housing only increases the FOV in the horizontal axis (perpendicular to its main axis). The FOV in the vertical axis is reduced in a similar way as a flat view-port. al housing only increases the FOV in the horizontal axis (perpendicular to its main axis). The FOV in the vertical axis is reduced in a similar way as a flat view-port. al interface has the advantage of allowing larger FOV (in one direction) for the five lateral cameras when compared with the option of having five individual flat ports (Figure 4) and is easier and less expensive to produce. The cylindrical housing only increases the FOV in the horizontal axis (perpendicular to its main axis). The FOV in the vertical axis is reduced in a similar way as a flat view-port. al housing only increases the FOV in the horizontal axis (perpendicular to its main axis). The FOV in the vertical axis is reduced in a similar way as a flat view-port.

For the top-looking camera (down-looking in water), the final design was a hemispherical piece. The manufacturing of this piece was significantly more challenging than a flat port alternative, since it required a thermoforming process to obtain the intended shape. However, the flat port option had to be discarded due to the severe reduction of the FOV that it would cause.

**Figure 5.**
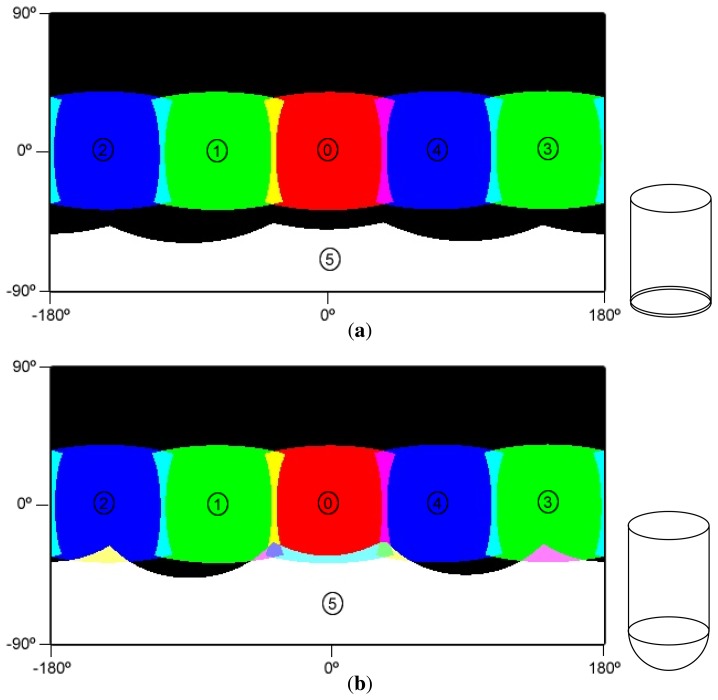
Equirectangular projection of the covered FOV at a 5-m distance in an underwater environment with different configurations. Each colored region represents the FOV of each camera (red, green, blue and white) and the areas of FOV intersection (other colors). (**a**) Projection of the covered FOV at a 5-m distance with a flat interface for the bottom camera; (**b**) projection of the covered FOV at a 5-m distance with a hemispherical interface for the bottom camera.

In order to test possible scenarios for the choices of the shape of the view-ports, a FOV simulator was implemented. This simulator uses ray-tracing techniques that take into account the full camera projection model and Snell's law for the refraction effects of the possible shapes of the housing. The results of the FOV simulator can be seen in Figure 5, which presents a full-sphere representation of the FOV, with a horizontal view angle in the *x*-axis of the plot and a vertical view angle on the *y*-axis.

**Figure 6.**
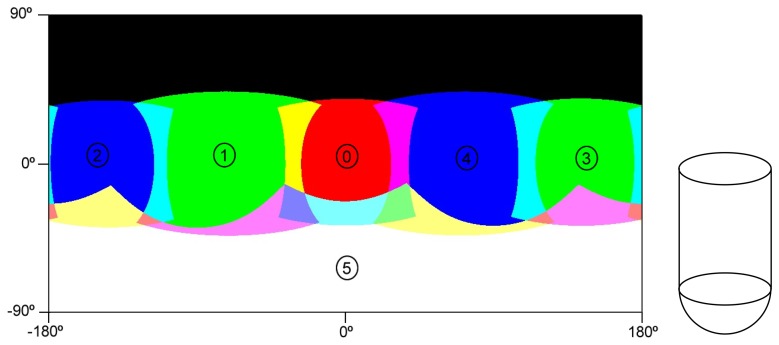
Equirectangular projection of the covered FOV at a 5-m distance with a hemispherical interface for the bottom camera and 2.95-mm focal length optics for Cameras 1, 4 and 5. Each colored region represents the FOV of each camera (red, green, blue and white) and the areas of FOV intersection (other colors).

As illustrated in Figure 5a, using a flat view port for Camera 5, this camera would not have any overlap with any of the other cameras. By contrast, the hemispherical view port allows some overlap, but not complete coverage of the lower hemisphere. For this reason, the original optics (3.3 mm of focal length) of Cameras 1, 4 and 5 were replaced for others with wider FOVs (2.95 mm of focal length) to achieve full coverage of the panoramic view (Figure 6). Further details on the computation of the FOV in underwater environments can be found in Sections 5 and 6.

During the design, it was important to ensure that the junction between the two parts of the dome was placed in a location that would not be visible by any camera, hence avoiding occlusions in the resulting image.

## 3. Single Camera Calibration

In this section, we first present the camera model used and then the calibration procedure for each one of the six individual cameras.

### 3.1. Camera Model

**Figure 7.**
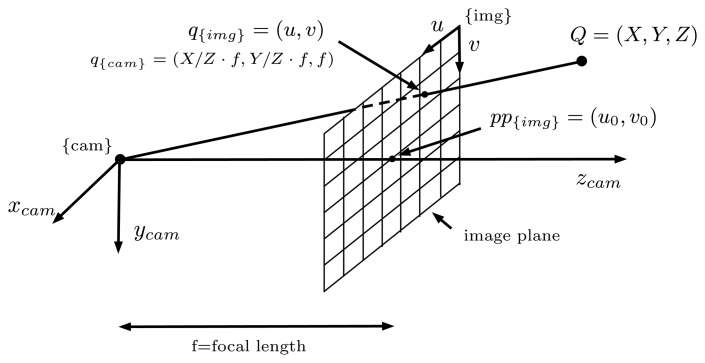
The pinhole camera model.

The pinhole camera model [8,9,24] has been adopted for this work due to its compactness (Figure 7) and accurate results. This model will allow one to project any 3D world point *Q* = (*X*, *Y*, *Z*) in the camera coordinate frame to a pixel position (*u*, *v*) in the image plane through Equations (1) and (2).

(1) $$\left[\begin{array}{c} u\\ v \end{array}\right]=\left[\begin{array}{cc} f_{x} & 0\\ 0 & f_{y} \end{array}\right]\;\left[\begin{array}{c} x\\ y \end{array}\right]+\left[\begin{array}{c} u_{0}\\ v_{0} \end{array}\right]$$

(2) $$x=\frac{X}{Z},y=\frac{Y}{Z}$$

where:

- (*u*_0_,*v*_0_) is the location of the principal point in the image plane coordinates. This is the point where the camera optical axis intersects the image plane and is normally located near the center of the image.
- (*f_x_*, *f_y_*) are the focal lengths along the *x* and *y* directions, expressed in pixels. Most cameras have sensors with squared pixels, where *f_x_* = *f_y_*.

**Figure 8.**
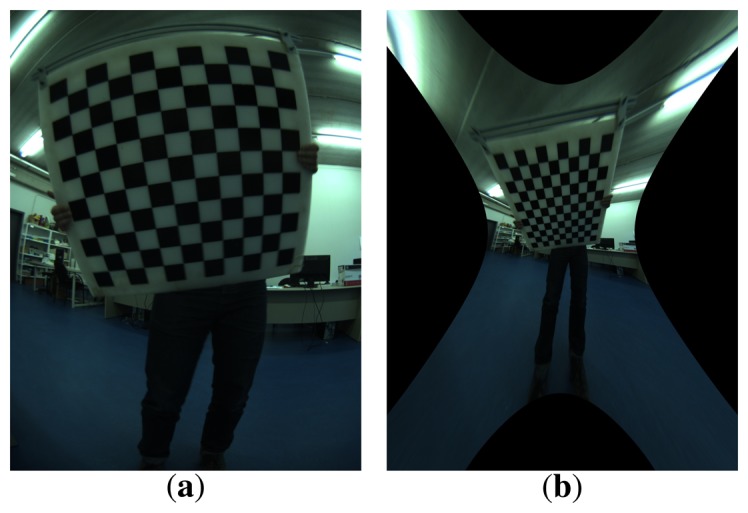
A sample image of a checker board captured by a wide-angle lens camera used for any standard calibration toolboxes, before (**a**) and after (**b**) the distortion correction. (a) Original image; (b) undistorted image.

However, all lenses induce image distortions that are not modeled by the pinhole camera model. The most common one is the radial distortion, which is due mainly to the shape of the lenses and produces nonlinear distortions along the radial direction from the principal point. The further from the center of the image, the higher is the radial distortion. Radial distortion is very strong for wide-angle lenses. For example, in Figure 8a, the edges of the rectangular board look curved when, in reality, they are perfectly straight. It can also be noticed in the image that this distortion becomes more important in the regions further from the center: the bottom edge of the board looks straighter than the top one. Fortunately there are models that introduce corrections in the original images (distorted) to undistort them and create a new undistorted (ideal) image that follows the pinhole model (Figure 8b). In this work, we adopt the model proposed by Kannala [10].

Let (u, v) be the ideal (non-observable, distortion-free) pixel image coordinates corresponding to a point *Q* = *X*, *Y*, *Z*. These coordinates do not match with the real ones (*u_d_*,*v_d_*) due to the distortion effect. The relation between the real point *x*, *y* (expressed in the camera reference plane, *Z* = 1) and a virtual point *x_d_*, *y_d_* that projects to the pixel coordinates (*u_d_*,*v_d_*) according to the pinhole model can be found through:

(3) $$\theta_{d}=\theta \left(1+k_{1}\theta^{2}+k_{2}\theta^{4}+k_{3}\theta^{6}+k_{4}\theta^{8}\right)$$

(4) $$x_{d}=x\left(\frac{\theta_{d}}{r}\right)$$

(5) $$y_{d}=y\left(\frac{\theta_{d}}{r}\right)$$

where *r*^2^ = *x*^2^ + *y*^2^, *θ* = *atan*(*r*) and *k*_1_, *k*_2_,…, *k_n_* are distortion coefficients.

### 3.2. Calibration

A two-step method for the calibration has been devised and implemented. In the first step, a standard calibration toolbox is used in order to provide an initial estimate of the intrinsic parameters. In the second step, these values are refined in order to obtain better results.

#### 3.2.1. Initialization

The OpenCV Library (Open Source Computer Vision Library) [25,26] methods for camera calibration and 3D reconstruction have been used to compute a first estimate of the intrinsic values of the six independent cameras. Given multiple shots of a planar grid (typically a checker board) acquired at different positions and orientations, the methods in this module allow one to compute the intrinsic camera parameters and extrinsic parameters for each of the views, based on the approaches presented in [10,24,27].

In most cameras, these methods are accurate enough for metrology applications, and there is no need for further refinement. However, for an OMS camera using wide-angle lenses, a refinement procedure can help to obtain better results, especially in the regions close to the borders of the image. In these regions is where the overlapping between camera images take place, and a very accurate calibration is required in order to avoid visible misalignments in the final panoramas.

#### 3.2.2. Refinement

**Figure 9.**
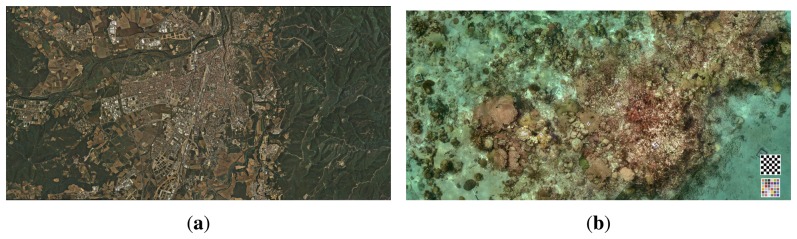
Posters used for the dry and underwater calibration, respectively. (**a**) Aerial image of the city of Girona used for both the intrinsic and extrinsic calibration procedures. The dimensions of the printed poster are 2.395 × 1.208 m; (**b**) Underwater image used for the optimization of the housing parameters. The printed poster measures 7.09 × 3.49 m and was placed in a flat area at the bottom of the test pool.

Due to the high distortion of the images (seen in Figure 8), it is not possible to place the checker board close to the corners of the image while seeing it entirely, as required by the standard calibration packages. This fact leads to calibration results that are inaccurate in the regions close to the image corners. For this reason, a different approach was implemented, which uses a more versatile pattern and allows results when only a portion of the pattern is visible in the images. An aerial image of the city of Girona (Figure 9a) has been used for this purpose, since it provides a large number of visual features at different scales.

Different shots of the poster in different positions and orientations have been taken, paying special attention to capturing parts of the poster rich in features in the corners of the images.

As a following step, scale-invariant feature transform (SIFT) [28] features are found in all of the selected shots and in the original poster image. Every feature has a keypoint and descriptor associated.

Afterward, putative matches between the features in the captured images and the original poster are found following Lowe's criterion [28]. Under this criterion, for every feature in the original image, the nearest neighbor in the captured image is defined as the keypoint with minimum Euclidean distance. In order to decide if this match is potentially the correct one, the second closest neighbor is found, and a ratio between its distance is computed as per Equation (6).

(6) $$\text{Ratio}=\frac{\text{Distance closest}}{\text{Distance second closest}}$$

If the ratio is smaller than a threshold, the matching is considered valid. The threshold chosen in this work was 0.7, which, according to Lowe, eliminates about 95% of false matchings and discards about 8% of the correct matches. To find the closest and the second closest matches among all of the features, the Fast Library for Approximate Nearest Neighbors (FLANN) [29] has been used to speed up the process, instead of purely brute force.

If the number of matches is greater than a fixed minimum, e.g., 100 matches, then the image is accepted to be used to optimize the intrinsic parameters. Otherwise, the image is rejected.

For the accepted images, the poster pose that minimizes the re-projection error of all correct matches found is estimated making use of the initial intrinsic parameters estimated in the previous step and solving the perspective-n-point (PnP) problem [9]. The PnP problem is the problem of the determination of the position and orientation of a calibrated camera given a set of *n* correspondences between 3D points and their 2D projections. In this case, the 3D points belong to the poster, and the 2D projections belong to the camera images. We assume that the poster is totally flat and in the plane *z* = 0; hence, the 3D points used are the coordinates of the matches in the original poster image, in the real scale, *i.e.*, meters, with its coordinate *z* set to zero. The 2D projections are the coordinates of matches in the camera images, in pixels. The implementation in OpenCV, which uses an iterative method to minimize the re-projection error, has been used to solve the problem. The solution is robust to outliers, thanks to the use of random sample consensus (RANSAC) [30]. The features discarded by RANSAC will not be used further in the intrinsic parameter optimization.

**Figure 10.**
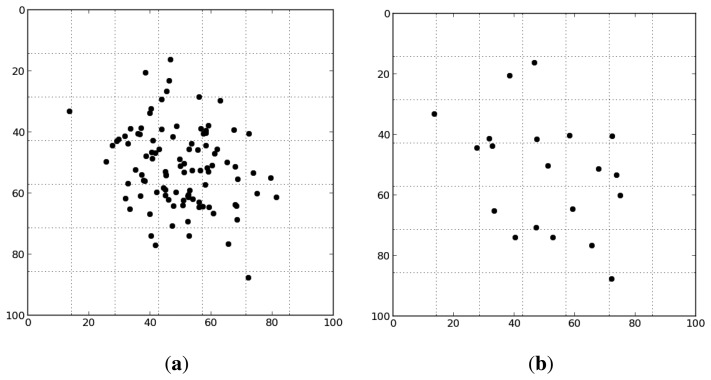
Selection of features with simulated data and 49 regions. (**a**) All feature matchings are associated with a region of the image; (**b**) only one feature per region is used for the optimization procedure.

From the set of all RANSAC inliers, a subset is chosen that contains image points that are well distributed on the image plane. The objective is to have approximately the same number of features in all regions of the image. A bucketing strategy [31] has been implemented to achieve this purpose. The image is divided into a number of disjoint regions of the same size. Then, every feature is associated with the region to which they pertain. Only one feature per region, selected randomly, will be used for the further calibration steps (Figure 10). The number of regions can be set arbitrarily, but must be in concordance with the size of the image and the number of features able to match in every shot, as it represents the maximum number of features selected. For this work, this parameter has been set to 500.

Finally, we are ready to define the optimization problem that will find the refined intrinsic parameters. The number of variables to estimate will be the totality of the intrinsic parameters (focal length, principal point coordinates and distortion parameters) and the pose of the poster (*x*, *y*, *z*, *α*, *β*, *γ*) in all of the selected images (Equation (7)). The initial values for the intrinsic parameters will be the ones found in the initialization step, while the initial values for the pose of the poster in every image will be the results of the PnP problem solved previously.

(7) $$\theta =\left[f,u_{0},v_{0},k_{1}\ldots k_{4},x_{img\left(0\right)},{\text{y}}_{img\left(0\right)},{\text{z}}_{img\left(0\right)},\alpha_{img\left(0\right)},\beta_{img\left(0\right)},\gamma_{img\left(0\right)}\ldots x_{img\left(n\right)},{\text{y}}_{img\left(n\right)},z_{img\left(n\right)},\alpha_{img\left(n\right)},\beta_{img\left(n\right)},\gamma_{img\left(n\right)}\right]$$

The re-projection error for every feature *p^p^* of the poster, which has a matching *p^c^* in a captured image, can be defined as:

(8) $$e_{\text{reproj}}\left(p^{p},p^{c},\theta \right)={\left\|\pi \left(p^{p},\theta \right)-p^{c}\right\|}^{2}$$

where *π* is the projection function of the 3D point associated with *p^p^* to the camera through Equations (1) and (3)–(5). For all of the matched features in an image, the total re-projection error can be expressed as:

(9) $$\sum_{k}e_{\text{reproj}}\left(p_{k}^{p},p_{k}^{c},\theta \right)$$

Then, the cost function that includes all of the selected images, can be expressed as:

(10) $$\sum_{img_{i}}\sum_{k}e_{\text{reproj}}\left(p_{k}^{p},p_{k}^{c},\theta \right)$$

This optimization problem is solved using a Levenberg–Marquardt algorithm [32,33] that minimizes the sum of all re-projection errors.

In order to quantify the uncertainty of the estimated parameters, a Monte Carlo analysis has been carried out. It consisted of repeating the same estimation procedure a significant number of times, but in this case, the set of paired features between the captured images and the original poster were not found using SIFT and RANSAC. For every feature in the original poster used for the estimation of the parameters, we find its projection in the captured image using the estimated intrinsic parameters and poster poses and add Gaussian noise. The Gaussian noise has zero mean, and the standard deviation was the value obtained as the standard deviation of all of the residues of the optimization. The study of the variability of the estimated parameters during the simulations provides valuable information about the uncertainty of the parameters. It also allows one to validate the adequacy of the input data in terms of the observability of the parameters being estimated. An example of this is checking that the used poster poses are sufficiently well spread to allow an accurate estimation of the parameters.

## 4. Extrinsic Calibration

**Figure 11.**
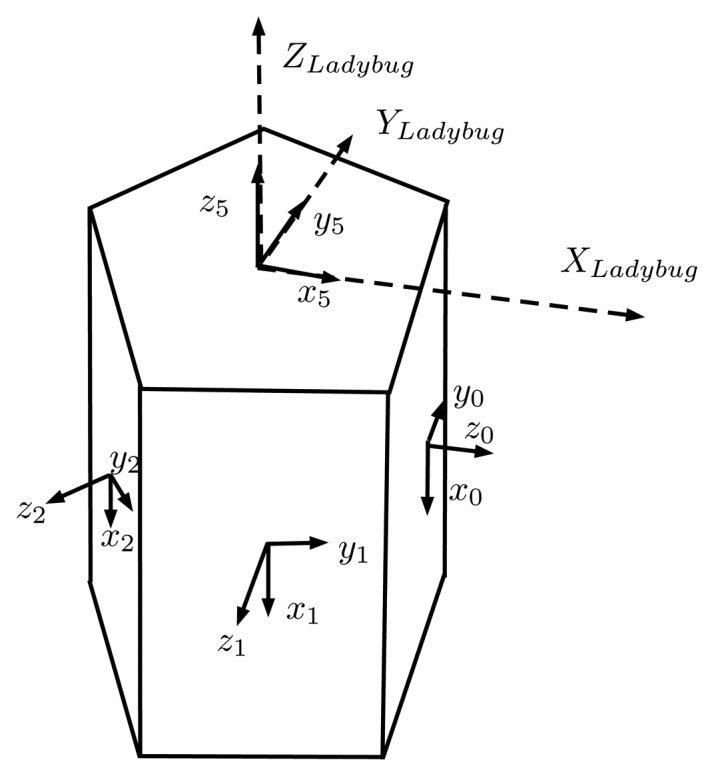
The relationship between cameras and the global coordinate system.

The main problem when working with multiple cameras is the determination of the exact geometric relationship between the different camera frames. These rotations and translations are referred to as the extrinsic parameters. In this work, both these and the intrinsic parameters are assumed not to change in time. Each camera has its own independent coordinate system, but the local coordinate system of Camera 5 has been defined as a global frame that will be used to deal with the external world (Figure 11). Therefore, the global coordinate system follows the standard convention for underwater frames, where the *z*-axis points downwards into the sea floor, making it more intuitive when integrating the camera in a robotic platform.

The procedure to estimate the extrinsic parameters will be very similar to the one used in the refinement of the intrinsic ones, in Section 3.2.2. However, in contrast, in this subsection, images from two or more cameras acquired at the same exact time frame are needed.

In order for the algorithm (Algorithm 1) to work properly, all cameras must acquire a recognizable section of the poster where one of the other cameras also acquires a different section of the poster simultaneously. The observation of different parts of the poster by two cameras at the same instant implicitly imposes constraints on the relative placement and relative orientation of the two cameras. These constraints are used in the cost function that is minimized. It should be noted that a standard checker calibration pattern cannot be used in this step, since it would be difficult to determine automatically which part of the pattern would be seen by each camera, due to the similarity of the squares in the grid. It is advantageous to have a wide range of images where the poster is visible from as many cameras as possible in different positions and orientations in order to estimate the unknown variables effectively.

**Figure 12.**
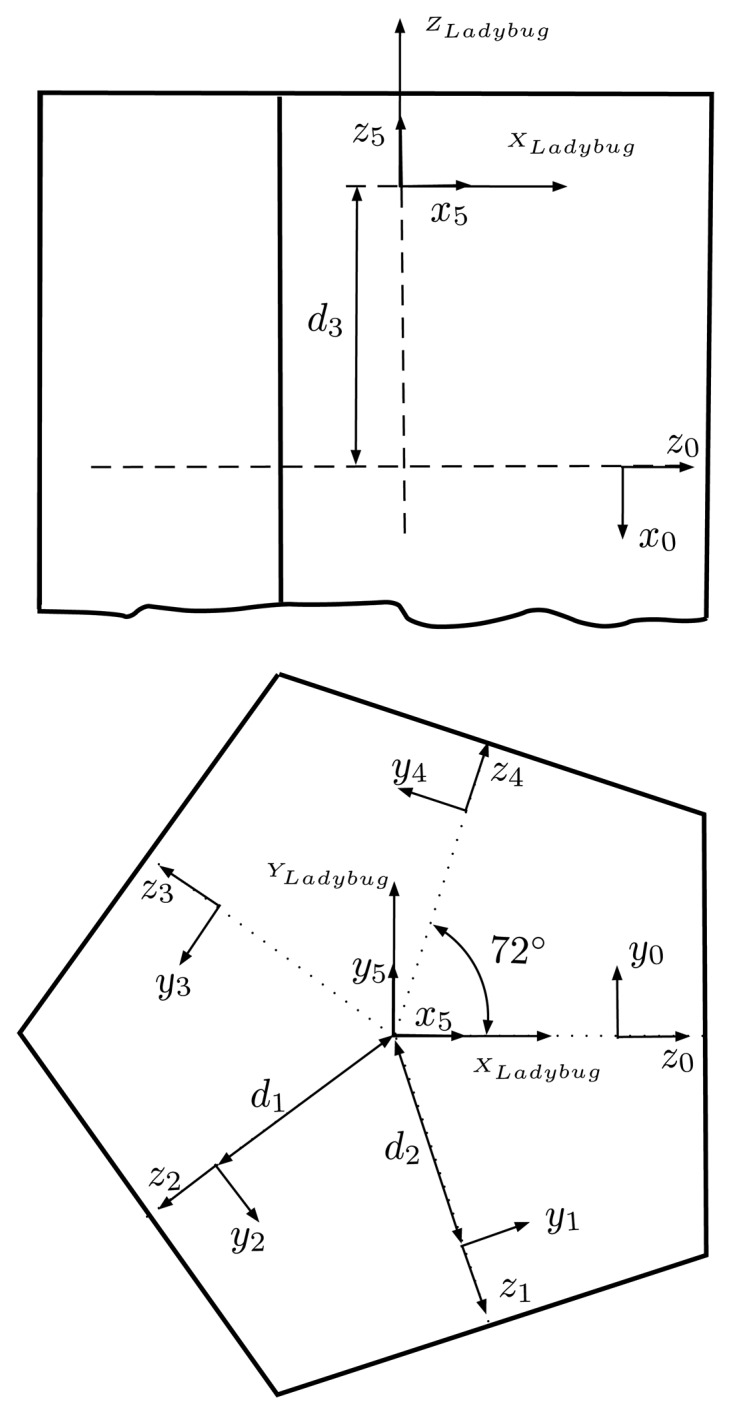
The geometrical unknowns during the first estimation of the extrinsic parameters of the cameras are: *d*_1_ (for Cameras 0, 2 and 3), *d*_2_ (for Cameras 1 and 4), *d*_3_ (for Camera 5) and the exact orientation of each camera. Side view (top) and top view (bottom).

In the first estimation, we will focus on estimating the rotations of each camera frame, and for that, we will assume that the sensors of the camera are placed ideally, *i.e.*, the optical centers of the five lateral cameras lie in the same plane and are placed in a perfect regular pentagon. The remaining camera is assumed to have its optical center placed along a perpendicular line passing through the center of the pentagon. With these assumptions, only three variables are needed to fully describe the location of the optical center of all of the cameras: *d*_1_ represents the distance between the center of the pentagon and the optical center of the three lateral cameras with the original optics (Cameras 0, 2, 3); *d*_2_ represents the same distance, but for the lateral cameras with the new 2.95-mm focal length optics (Cameras 1, 4); and *d*_3_ represents the minimum distance from the plane containing the lateral cameras to the optical center of the remaining camera (Figure 12). For the optimization, the initial values of these three variables will be an approximated physical measurement.



**Algorithm 1** Extrinsic parameter calibration.
 Find SIFT features in original poster image **for**
*frame* = 0 to *frame* = *n*
**do**  **for**
*cam* = 0 to *cam* = 5 **do**   Find SIFT features in the image   Poster found?  **end for**  **if** Poster found in more than two cameras **then**   Solve PnP problem for one of the cameras   Find initial estimation of poster position in global coordinates   Add to the list of frames and features to be used in the optimization  **end if** **end for** Optimize parameters to minimize re-projection error sum


The orientation of the global and the Camera 5 frames will be fixed, while the orientation of frames of Cameras 0 to 4 will be estimated from the optimization procedure. Its initialization will be its ideal orientation (Figure 12). For Cameras 0 to 4, each camera is rotated 72° along the *x*-axis with respect to its neighbors, with the *z*- and *y*-axis of Camera 0 coinciding with the *x*- and *y*-axis of the global frame.

An initial pose of the poster for every different time frame will be needed to start the optimization procedure. This can be estimated by solving the PnP problem from any camera seeing the poster in that exact time frame and then converting it to global coordinates.

The vector containing the totality of parameters to estimate will be:

(11) $$\theta =\left[d_{1},d_{2},d_{3},\alpha_{c\left(1\right)},\beta_{c\left(1\right)},\gamma_{c\left(1\right)}\ldots \alpha_{c\left(5\right)},\beta_{c\left(5\right)},\gamma_{c\left(5\right)},{\text{x}}_{f\left(0\right)},{\text{y}}_{f\left(0\right)},{\text{z}}_{f\left(0\right)},\alpha_{f\left(0\right)},\beta_{f\left(0\right)},\gamma_{f\left(0\right)}\ldots {\text{x}}_{f\left(n\right)},{\text{y}}_{f\left(n\right)},{\text{z}}_{f\left(n\right)},\alpha_{f\left(n\right)},\beta_{f\left(n\right)},\gamma_{f\left(n\right)}\right]$$

where [*α_c_*_(_*_i_*_)_, *β_c_*_(_*_i_*_)_, *γ_c_*_(_*_i_*_)_] represent the orientation of camera *i* and [*x_f_*_(_*_j_*_)_, *y_f_*_(_*_j_*_)_, *z_f_*_(_*_j_*_)_, *α_f_*_(_*_j_*_)_, *β_f_*_(_*_j_*_)_, *γ_f_*_(_*_j_*_)_] represent the pose of the poster position in the global frame in time frame *j*.

Through Equations (8)–(10), the cost function that includes the re-projection errors of all of the features present in the images of the selected frames can be expressed as:

(12) $$\sum_{{\text{frames}}_{j}}\sum_{{\text{cam}}_{i}}\sum_{k}e_{\text{reproj}}\left(p_{k}^{p},p_{k}^{c},\theta \right)$$

After this first estimation, a new optimization procedure will be performed, fixing this time the rotations of the camera frames and without constraints regarding its location. The cost function will remain the same, while the vector containing the totality of parameters to estimate will be:

(13) $$\theta =\left[{\text{x}}_{c\left(1\right)},{\text{y}}_{c\left(1\right)},{\text{z}}_{c\left(1\right)}\ldots {\text{x}}_{c\left(5\right)},{\text{y}}_{c\left(5\right)},{\text{z}}_{c\left(5\right)},x_{f\left(0\right)},{\text{y}}_{f\left(0\right)},{\text{z}}_{f\left(0\right)},\alpha_{f\left(0\right)},\beta_{f\left(0\right)},\gamma_{f\left(0\right)}\ldots x_{f\left(n\right)},{\text{y}}_{f\left(n\right)},{\text{z}}_{f\left(n\right)},\alpha_{f\left(n\right)},\beta_{f\left(n\right)},\gamma_{f\left(n\right)}\right]$$

where [*x_c_*_(_*_i_*_)_, *y_c_*_(_*_i_*_)_, *z_c_*_(_*_i_*_)_] represent the position of camera *i*.

The estimation of the relative rotations and translations of the cameras is done in two stages due to a unique step that led to physical non-sense values for the translations due to the sensitivity of the rotation parameters. A Monte Carlo analysis has been carried out to determine the uncertainty of the estimated parameters, similarly to the one described for the intrinsic parameters estimation.

## 5. Underwater Calibration

The direction of the rays of light changes in every medium transition found along the path from a point underwater to the imaging sensor inside the camera. In order to model accurately the distortion due to this effect, it becomes essential to explicitly model and simulate the intersection of each light ray with different media, as detailed next.

### 5.1. Ray Tracing

Once the intrinsic parameters of each camera are known, each pixel of an undistorted image can be associated with a 3D ray originated at the optical center of the camera and described in the 3D coordinate frame of the camera. The direction vector of this ray can be computed through Equation (14).

(14) $$v_{0\left\{\text{local}\right\}}=\left(\frac{u-u_{0}}{f_{x}},\frac{v-v_{0}}{f_{y}},1\right)$$

The local vector can be transformed to the global frame pre-multiplying by the rotation matrix *R* that relates both coordinate systems:

(15) $$v_{0}=R\cdot v_{0\left\{\text{local}\right\}}$$

Let *p*_0_ be the optical center of one of the cameras. The 3D ray can be described as:

(16) $$s=p_{0}+kv_{0}$$

where *k* is a scalar.

**Figure 13.**
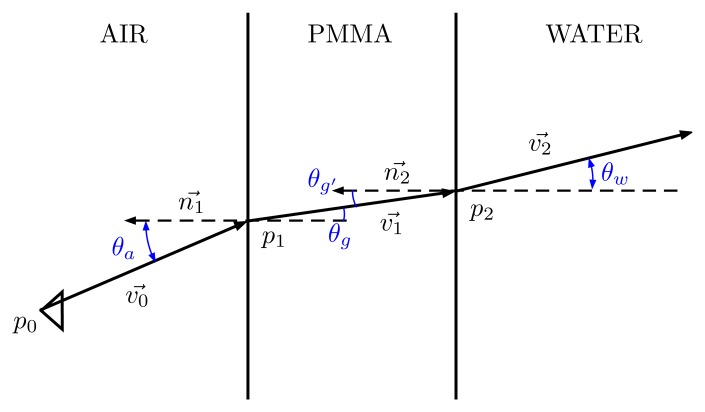
Ray tracing schematic of a single optical ray passing through air, PMMA and water.

When the camera is inside the waterproof housing, this ray will change its direction when transitioning to the PMMA interface, due to the refraction effect. The direction of the refracted ray, *v_r_*, can be computed through Snell's law. In a 2D plane, as Figure 13, Snell's law can be expressed as:

(17) $$\sin\left(\theta_{a}\right)\cdot n_{\text{air}}=\sin\left(\theta_{g}\right)\cdot n_{\text{PMMA}}$$

where *θ_a_* is the angle between the incident ray and the normal vector of the surface in the intersection point, *θ_g_* is the angle between the refracted ray and the normal vector and *n_air_*, *n_PMMA_* are the refractive indexes of air and PMMA, respectively.

Due the complex geometry of the dome, it is better to work in the 3D space and use the following expression of Snell's law:

(18) $$v_{1}=\frac{n_{\text{air}}}{n_{\text{PMMA}}}\left(n_{1}\times \left(-n_{1}\times v_{0}\right)\right)-n_{1}\sqrt{1-{\left(\frac{n_{\text{air}}}{n_{\text{PMMA}}}\right)}^{2}\left(n_{1}\times v_{0}\right)\cdot \left(n_{1}\times v_{0}\right)}$$

where *n*_1_ is the normal vector of the surface in the intersection point between the ray and the surface and *v*_0_, *v*_1_ are the direction vectors of the incident and refracted ray.

In order to find both *p*_1_ and *n*_1_, the waterproof housing must be geometrically modeled. It can be expressed as the union of a cylinder of radius *r*, direction vector *v_c_* and origin *p_c_*, with a hemisphere of radius *r* and center *p_s_*.

The points *q* of the cylinder can be expressed as:

(19) $$\left\|\left(q-p_{c}\right)-\left(v_{c}\cdot \left(q-p_{c}\right)\right)v_{c}\right\|=r$$

The points *q* of the hemisphere can be expressed as:

(20) $$\left\|q-p_{s}\right\|=r$$

Knowing both the expression for the optical ray and the geometrical model of the housing, we can find the intersection point by replacing the expression of the ray by the surface point *q*, both in the case of the cylindrical part:

(21) $$\left\|\left(p_{0}+kv_{0}-p_{c}\right)-\left(v_{c}\cdot \left(p_{0}+kv_{0}-p_{c}\right)\right)v_{c}\right\|=r$$

(22) $${\left(p_{0}-p_{c}+kv_{0}-\left(v_{c}\cdot \left(p_{0}-p_{c}+kv_{0}\right)\right)v_{c}\right)}^{2}-r^{2}=0$$

and the hemispheric:

(23) $$\left\|p_{0}+kv_{0}-p_{s}\right\|=r$$

(24) $${\left(kv_{0}\right)}^{2}+2k\left(v_{0}\cdot \left(p_{o}-p_{s}\right)\right)+{\left(p_{0}-p_{s}\right)}^{2}-r^{2}=0$$

Solving the value of *k* from these equations, the intersection point can be found as:

(25) $$p_{1}=p_{0}=kv_{0}$$

Before applying Snell's law, the normal vector of the surfaces in the intersection point *p*_0_ needs to be found. For the cylindrical part, the normal in a point *q* can be found through:

(26) $$n=p_{c}-q+\left(\left(p_{c}-q\right)\cdot v_{c}\right)v_{c}$$

while the normal vector for the hemispheric part in a point *q* can e found through:

(27) $$n=p_{s}-q$$

We can finally compute the refracted ray direction vector *v*_1_ through the vectorial expression of Snell's law (Equation (18)). The refractive indexes of air and PMMA are assumed to be invariant for the conditions in which the camera will work. The assumed values are *n_air_* = 1 [34] and *n_PMMA_* = 1.4914 [35] (for wavelengths λ = 0.589 (μm). The refracted ray can be expressed as:

(28) $$s=p_{1}+kv_{1}$$

This ray will change once again its direction when moving from PMMA to water. The new refracted ray can be computed in an analog way to the transition detailed above. The refractive index of water for this transition has been assumed as *n_water_* = 1.333 (λ = 0.589 (μm). This is the refractive index for fresh water at 20° [36], which was approximately the water conditions of the water tank when the images for the calibration were taken. In the case of seawater, the refractive increases slightly with the salinity. The value of the refractive index can be easily tuned for further use after the calibration procedure if required.

Knowing both the intrinsic and extrinsic parameters of all of the cameras and through the use of the ray tracing approach, it is possible to project any 3D point underwater to any of the cameras composing the omnidirectional image. However, this projection is not straightforward. The distance *d* from the camera to the 3D point will be computed, and through the ray tracing study and an iterative method, it is possible to find the projection of the 3D point into the camera. An initial value for the projected pixel is required, and then iterating its position until the error between the 3D point and the point of the ray associated with the pixel at a distance *d* is negligible. The initial value of the pixel can be initialized with the value of the projection in air or simply to the center of the image. This iteration procedure can be solved through the Levenberg-Marquardt algorithm.

### 5.2. Housing Parameters Optimization

Due to the ray bending, any small variation in the assumed relative position of the housing can significantly affect the final direction of the rays and end up generating projection errors. In order to avoid this, the difference between the estimated relative position and the real one has to be minimized. There is no guarantee that the center of the global camera frame coincides exactly with the cylinder axis of the housing, nor is the alignment between the global coordinate system and the cylinder perfect. For this purpose, the relative position of the housing with respect to the camera will be estimated in a procedure almost identical to the one in Section 4, but using now images captured underwater and with a different poster specially prepared to be placed underwater (Figure 9b). The poster was placed at the bottom of a water tank of 5 m in depth, and different images were captured with the camera in different positions and orientations. It is important that the images are captured in the best lighting conditions possible, in order to minimize the light absorption caused by water, making difficult the recognition of features on the images.

**Figure 14.**
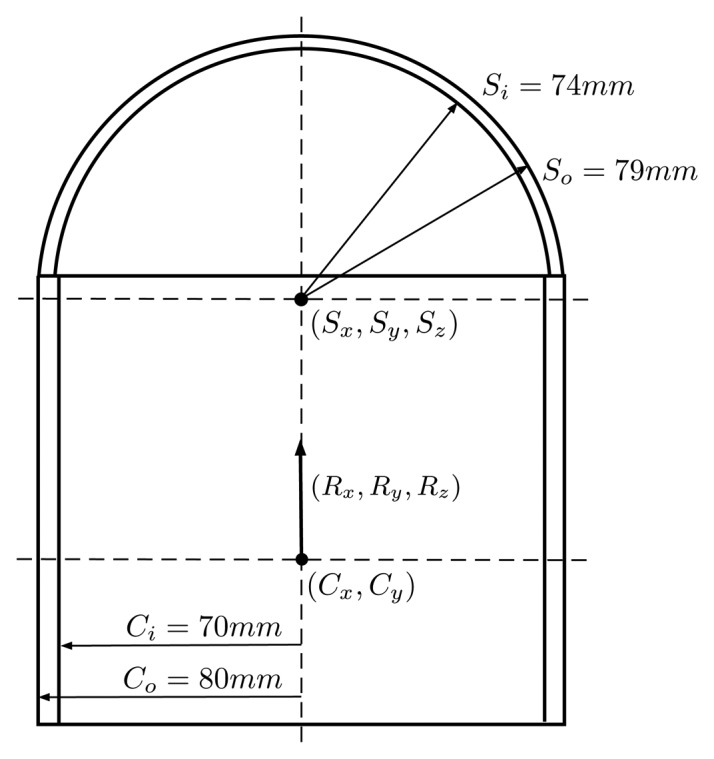
Cross-section representation of the PMMA waterproof housing.

The parameters to optimize (Equation (29)) are the location of the center of the cylinder (*C_x_*,*C_y_*), the orientation of the cylinder (*R_x_*, *R_y_*, *R_z_*) and the position of the center of the hemisphere (*S_x_*, *S_y_*, *S_z_*), illustrated in Figure 14, apart from the poses of the poster for every different time frame *i* used [*x_f_*_(_*_i_*_)_, *y_f_*_(_*_i_*_)_, *z_f_*_(_*_i_*_)_, *α_f_*_(_*_i_*_)_, *β_f_*_(_*_i_*_)_, *γ_f_*_(_*_i_*_)_]. All of the parameters are with reference to the global frame of the camera.

(29) $$\theta =\left[C_{x},C_{y},R_{x},R_{y},S_{x},S_{y},S_{z},x_{f\left(0\right)},y_{f\left(0\right)},{\text{z}}_{f\left(0\right)},\alpha_{f\left(0\right)},\beta_{f\left(0\right)},\gamma_{f\left(0\right)}\ldots x_{f\left(n\right)},y_{f\left(n\right)},z_{f\left(n\right)},\alpha_{f\left(n\right)},\beta_{f\left(n\right)},\gamma_{f\left(n\right)}\right]$$

The initial values for the target parameters are established from the knowledge of approximate geometry of the housing. For the poster pose initialization, for every different time frame, the poster pose can be estimated by solving the PnP problem from any camera seeing the poster without taking into account the distortion caused by the housing. It is preferably to compute this initial pose from Camera 5, as the distortion caused by the hemispherical port is less important than that caused by the cylindrical port; hence the initialization values will be more accurate. The estimation process is similar to the one described in Section 4. A cost function is defined on the residues of the re-projection of the points detected in the multiple images of the poster (Equation (12)). This cost function is parametrized by the unknowns described above and minimized using the Levenberg–Marquardt algorithm. A Monte Carlo method is also used to determine the uncertainty of the estimated values, as described in the last section.

## 6. Results

In this section, we present both the numerical and graphical results of all of the steps during the calibration procedure. In order to interpret correctly the reconstructed panoramas, a subsection is first presented explaining in detail the process behind the creation of the panoramas.

### 6.1. Panorama Composition

After a successful calibration of the omnidirectional camera, each pixel of any image can be associated with a 3D ray in space. The length of this ray depends on the distance to the objects in the scene. Except for the small area where there is image overlap, it is not possible to estimate the distance to the objects from just a set of images acquired at a single location. For the overlapping parts, it is possible to estimate the distance to features seen from both cameras using the same method as in a conventional stereo camera. However, this would be very difficult in underwater environments where the overlapping is very small, the baseline is extremely short and there are very few features in most of the environments. Furthermore, this would be expensive computationally and would make it impossible to render panoramic images in real time with the existing hardware. For this reason, for visualization purposes, the world around the camera is assumed to be a sphere, where all of the points sensed by the camera are at a constant distance, pre-selected by the final user. Once the sphere radius is defined, a spheric point cloud is quick to compute, and it can be easily loaded in a computer 3D viewer or re-projected into a 2D image. For omnidirectional cameras, the equirectangular projection is the most commonly used [37].

**Figure 15.**
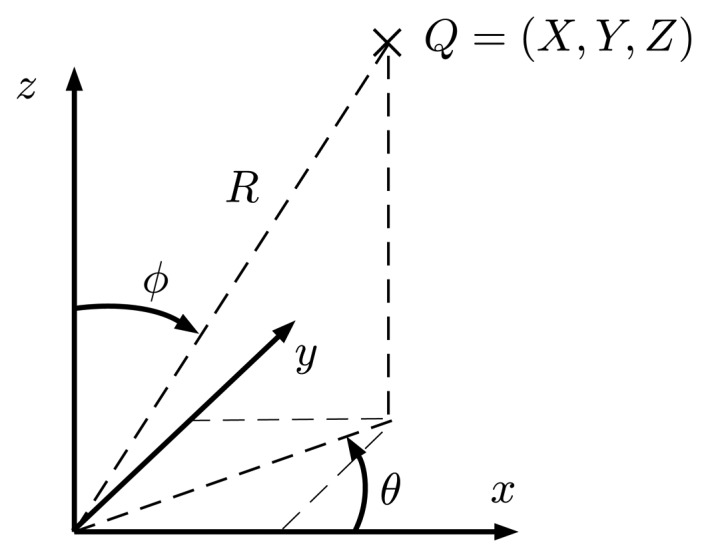
Conversion from Cartesian to spherical coordinates.

Given a world point in Cartesian coordinates *Q* = (*X*, *Y*, *Z*), it can be converted to spherical coordinates (Figure 15) *Q* = (*θ*, *ϕ*, *R*) through Equations (30)–(32).

(30) $$R=\sqrt{X^{2}+Y^{2}+Z^{2}}$$

(31) θ=atan2(Y,X),0≤θ≤2π

(32) $$\phi =a\cos\left(\frac{Z}{R}\right),0\leq \phi \leq \pi$$

**Figure 16.**
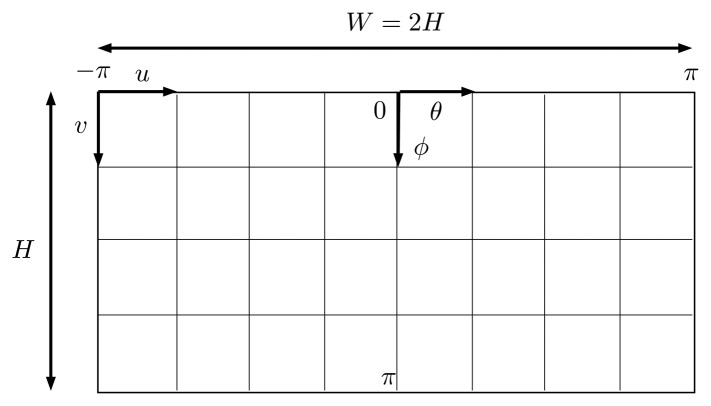
Equirectangular projection.

The equirectangular projection projects a given point *q* to a cylinder (Figure 16) through Equations (33) and (34):

(33) $$u=\frac{\theta +\pi }{2\pi }\cdot W$$

(34) $$v=\frac{\phi }{\pi }\cdot H$$

The inverse equations are:

(35) $$\theta =\frac{u\cdot 2\pi }{W}-\pi$$

(36) $$\phi =\frac{v\cdot \pi }{H}$$

The first step when composing a panorama (Algorithm 2) is choosing its parameters: projection type, projection distance and final size. For every pixel of the panorama, the 3D point it represents is computed according to the inverse equations of its projection (Equations (35) and (36)). This 3D point is then projected to each one of the six cameras according to its intrinsic and extrinsic parameters. For underwater panoramas, it will be necessary to do a numeric iteration to find this projection through the equations presented in Section 5. If the point is only in the FOV of one camera, we will give to the pixel of the panorama the same intensity values as the pixel corresponding to the projection of the 3D point into the camera. In the case of overlapping regions, a blending criterion [38] will be needed, to establish the value of the panorama pixel.



**Algorithm 2** Panorama construction.
 Choose projection Set suitable sphere radius: r Set panorama size **for** all pixels in panorama **do**  Compute 3D point according to pixels position, projection inverse equations and r  **for**
*cam* = 0 to *cam* = 5 **do**   Project 3D point to camera image.   **if** Point falls into camera FOV **then**    Store color information and location of the pixel.   **end if**  **end for**  **if** The point is only seen for one camera **then**   Give to the pixel the same value of the pixel of the camera that represents the point  **else if** The point is seen for more than one camera **then**   Give to the pixel a value according to a selected blending criterion  **else** {The point is not in the FOV of any camera}   Set the value of the pixel to black  **end if** **end for**


Three different criteria are presented below. The first one does not do any smoothing on the transition between cameras. The second one is a basic, but fast smoothing approach, which can be executed online. The last one makes a smooth blending offline. A practical comparison between them can be found in Table 1.

- Closest camera: From all of the pixels that represent the same 3D point:
  (37) $$\begin{array}{cc} p_{\text{cam}}=\left(u_{\text{cam}},{\text{v}}_{\text{cam}}\right), & 0\leq \text{cam}\leq 5 \end{array}$$
  the one with the minimum euclidean distance to its principal point is chosen:
  (38) $$d\left(p_{\text{cam}},pp_{\text{cam}}\right)=\sqrt{{\left(u_{\text{cam}}-u_{0,\text{cam}}\right)}^{2}+{\left(v_{\text{cam}}-v_{0,\text{cam}}\right)}^{2}}$$
  The reason for using this criterion is that the distortion is lower for the points closer to the principal point of the image than for the ones further away. This will reduce the error in the panoramas related to the distortion of the lenses.
- Weighted mean: A weighted mean of all of the pixels representing the same 3D point is performed, giving more weight to the pixels closer to its principal point. A blending width threshold will decide where this criterion is applied. A bigger blending width will mean a smoother transition, but with a higher degree of blurriness. This method can be considered as a simpler version of Burt and Adelson's method [39].
  The minimum distance between the pixels and their principal point is computed using Equation (38). The final value of the intensity for each one of the channels (R,G,B) is defined as:
  (39) $$I_{\text{channel}}=\frac{\underset{\text{cam}}{\text{∑}}I_{p,\text{cam},\text{channel}}\cdot \left(\text{blending width}-\left(d\left(p_{\text{cam}},pp_{\text{cam}}\right)-d_{\min}\right)\right)}{\underset{\text{cam}}{\text{∑}}\text{blending width}-\left(d\left(p_{\text{cam}},pp_{\text{cam}}\right)-d_{\min}\right)}$$
  for *blending width* ≠ 0.
- Gradient blending: Gradient blending methods are able to unify different exposures seamlessly and can lead implicitly to a high dynamic range from a set of low dynamic range images. However, they require solving large sparse systems of equations to recover the luminance from the gradient vectors. This method is based on the computation of the vertical and horizontal image gradients of the unblended image produced by the first criterion. The gradient field is modified to impose null gradients along the transition borders among images. This modified gradient field is no longer consistent, in the sense of allowing one to recreate an image that has this exact gradient field. Therefore, a least squares approximation to a consistent gradient field is performed. The final blended image is obtained from this approximated gradient field. More details for this technique can be found in [38,40].

To improve the final panoramic image and make the transitions between cameras softer, a gain compensation can be carried out. When in capture mode, the camera itself computes overall gain and shutter values to obtain visually pleasant images. All of the individual cameras are set with these values, but due to the fact that the lighting conditions are different for each one of the cameras and the replaced optics (Section 2) have slightly different aperture values than the original ones, there are luminosity differences between images from different cameras. To reduce these differences, the approach presented by Brown and Lowe [41] was implemented. This technique uses an individual gain for each image to minimize the intensity differences on the overlapping regions between images. The results of applying this technique, compared with the images without correction, can be seen in Table 1.

For a correct visualization of the results, it is very important that the estimated “radius” where the image is projected is properly set. It is important to notice, as well, that if all of the objects of the scene are not at the same distance, it will not be possible to align perfectly different images composing the panorama. This is not an error of the projection of the images, but rather an effect of the unknown distance of all of the objects of the scene, which is assumed to be constant and fixed. As can be seen in Table 2 for a scene with objects at a different distance, different distance projections will result in different objects aligning the overlapping regions of the panoramas.

### 6.2. Single Camera Calibration

As explained in Section 2, for achieving a complete semi-spherical FOV without blind spots, two different type of lenses have been used. Three cameras were left with the original optics of a 3.3-mm focal length, while the other three were replaced with wider angle lenses of 2.95 mm in focal length. For the sake of simplicity, we will present here only the results for one of the optics, namely a 2.95-mm one, since it is more illustrative due to the presence of stronger distortions. The results for the other optics are similar, apart from minor numeric variations.

For the initialization step, where a standard calibration toolbox has been used, a few parameters need to be set before executing the toolbox, to obtain the best results possible. These parameters are the initial guesses for focal lengths and the principal point location. The initial guesses were set from the information provided by the lenses and camera manufacturers. The numeric index used to evaluate how accurate the calibration is is the root mean square (RMS) of re-projection error defined in Equation (40). The final RMS was 0.479 px for Camera 5 (2.95-mm focal length).

(40) $$\text{RMS}\left(\text{reprojection error}\right)=\sqrt{\frac{\sum d{\left(x_{i},{\hat{x}}_{i}\right)}^{2}}{n}}$$

Although the results in the first step appear to be very good, it is worth noting that these calibrations are only done with data from the image center, where all of the squares of the calibration grid are visible. Therefore, it is difficult to evaluate the calibration quality on the image corners, where the distortion effects are most prominent. For omnidirectional cameras, a very accurate single camera calibration is required. The refinement step will use a larger number of features and is better distributed, thus allowing one to obtain more accurate results.

**Figure 17.**
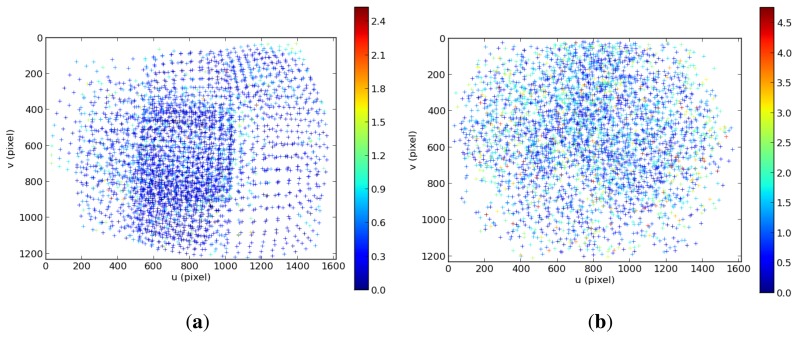
Comparison between the location of the features and its re-projection error in the initialization (**a**) and refinement step (**b**) for the intrinsic calibration of Camera 5 (2.95-mm focal length).

**Figure 18.**
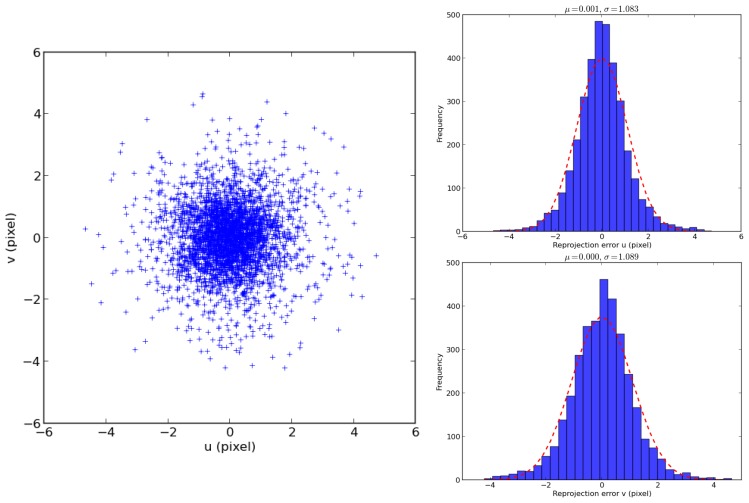
Re-projection error of the features after the refinement step of Camera 5.

The initial values for the optimization procedure are the values found by the standard calibration toolbox in the previous step. Table 3 presents the final results of the calibration. During the optimization, the RMS decreased from an initial value of 1.35 px to 1.09 px. The first column shows the result of the standard calibration and the second column the results of the refinement step. Thanks to the refinement step, it has been possible to find matches in regions where it was not possible using only the checker board and the standard calibration methodology (Figure 17). This allowed one to estimate the intrinsic parameters much more accurately (Table 3). This is due to the fact that the dataset used in the refinement step had much better distributed features all around the image than the checkerboard dataset, which has most of its features in the central region of the image where the distortion is less prominent. As can be seen in Figure 18, the distribution of the errors closely follows the shape of a 2D Gaussian probability distribution. The result of the focal length after the refinement procedure is much more similar to the values provided by the lense manufacturers than after the initialization step.

The last column of Table 3 shows the standard deviation of a Monte Carlo analysis with 1000 runs where it can be seen that the focal length, principal point and distortion coefficients are well determined.

### 6.3. Extrinsic Calibration

Once the intrinsic parameters of all of the cameras are known, the next step is the determination of the external geometric relationship, *i.e.*, translation and rotation, between them. Coarse physical measurements have been used for the initial values of *d*_1_, *d*_2_ and *d*_3_ (Figure 12). For the rotations, the ideal values have been used. The numeric results obtained during the optimization of the extrinsic parameters can be seen in Table 4. As in the intrinsic parameter calibration, the re-projection error of the features follows a Gaussian distribution and does not depend on its location on the image. The last column of Table 4 shows the standard deviation registered during the Monte Carlo analysis with 1000 runs. As can be seen, the estimated parameters are well determined and show low uncertainty.

**Figure 19.**
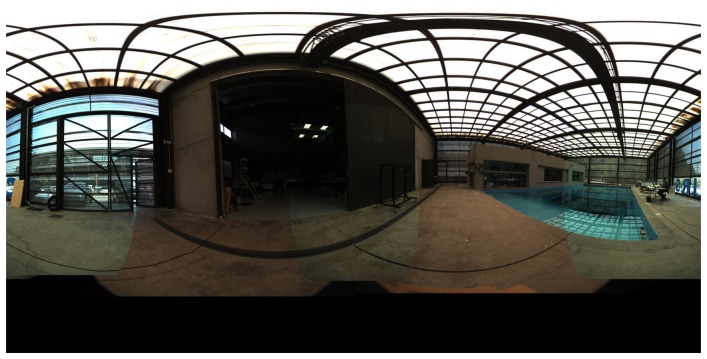
Equirectangular projection of the interior of the CIRS building, created with a re-projection distance of 10 m and using the closest blending method.

Excellent graphic results have been obtained using this calibration parameter, as can be seen in Figure 19.

### 6.4. Underwater Housing Optimization

The last stage of the calibration consists of the estimation of the parameters associated with the geometry of the waterproof housing that cannot be measured directly. The starting values for these parameters in the optimization are given by approximated physical measurements. The numeric results obtained during the optimization can be seen in Table 5. The distribution of the errors approximately follows the shape of a 2D Gaussian probability distribution with a 3.45-pixel standard deviation.

**Figure 20.**
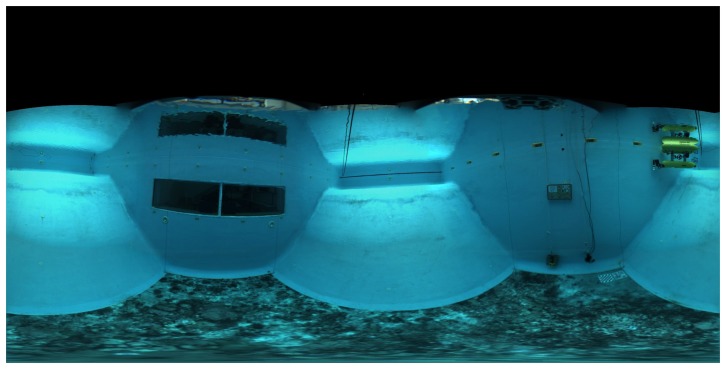
Equirectangular panorama of the CIRS water tank projected at a distance of 4 m with gradient blending.

Good visual results have been obtained using these calibration parameters (Figure 20). Misalignments between camera transitions are barely visible when the panoramas are rendered at the correct distance, even with the simplest blending criterion. Even though, the numeric results obtained are not as flawless as in the previous steps. The three main causes analyzed that could have a negative impact on the mean re-projection error obtained are as follows:

1. The hemispherical part of the housing cannot be modeled as a perfect hemisphere. Given the shape and dimensions of the camera system, it is not feasible to manufacture the PMMA cover as a single piece. For this reason, the hemispherical dome was thermoformed separately and then attached with adhesive to a prefabricated cylindrical body. Although this solution is simple and inexpensive, the hot-forming process is dimensionally inaccurate and induces changes in the thickness of the material along the body of the dome. During the ray tracing, this part has been considered as a perfect hemisphere, which may induce inaccuracies in both the intersection point between the ray and the hemisphere and the refracted ray direction. New manufacturing techniques and a new housing design are being studied to improve this condition.
2. The poster used during the estimation of the underwater parameters was placed at the bottom of a water tank. Due to the upward force that the water under the poster applies against it, it is not possible to guarantee the exact flatness of the poster, especially in its corners. This fact could have increased the final RMS due to the introduction of inaccuracies in the location of the features in the estimation procedure.
3. The refraction indexes used for the estimation of the housing parameters could not have been accurate enough according to the environmental conditions, leading the optimization algorithm to a higher residual re-projection error.

## 7. Conclusions

In this paper, we have presented in detail a complete method to calibrate and model an underwater omnidirectional camera composed of multiple single cameras (OMS). This calibration has three different stages. The first one estimates the intrinsic parameters of the individual cameras composing the OMS and can handle strong distortions introduced by wide-angle lenses. The second stage estimates the geometrical relationship between all of the individual frames with respect to a global frame and can deal with cameras with small or no overlapping between cameras. The third stage consists of a ray tracing approach to model correctly the light rays when the camera operates inside a housing in underwater environments. This study can be easily modified to be adapted to different housing geometries. Using this approach, an FOV simulator was developed and used to determine a suitable housing shape and optics replacements to cover a complete hemisphere when operating underwater. The final solution for the Ladybug 3 camera required the replacement of three of the original optics for others with wider FOVs and a dome-shaped housing composed of a cylindrical and a hemispheric part. The results, both numerical and graphical, are very good for the dry calibration part. The parameters estimated have low uncertainty, and the RMS of the re-projection error is small (1.35 px). The final panoramas obtained have very good quality, and there are no visible misalignments when rendered at the correct distances. The results for underwater operation present good results, even though the RMS of the re-projection error is larger (3.85 px), mainly due to unmodeled imperfections from the manufacturing of the hemispherical section of the housing. As future work, other housing shapes and manufacturing techniques will be further analyzed in order to reduce the amount of distortion introduced by it and to improve the results. The graphical results obtained are very good, and misalignments not due to the rendering distance are barely visible.

The high quality of the overall results validate the approach and the methods proposed and pave the way for this OMS to be used both for visualization purposes as a means for popular science or dissemination or as an additional sensor in AUVs and ROVs for navigation, mapping and sea exploration.

## Figures and Tables

**Table 1.** Transition in a panorama with different blending criteria applied and without or with individual gain correction. The color transition is more homogeneous when applying gain corrections, and the transition is smoother when moving from left to right using the blending criterion approach.

	**Blending Criterion**		
	**Closest Camera**	**Weighted Mean**	**Gradient Blending**
Original color	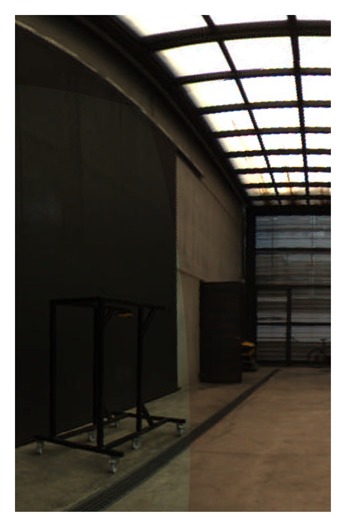	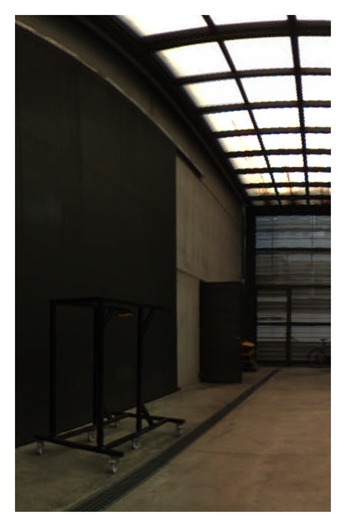	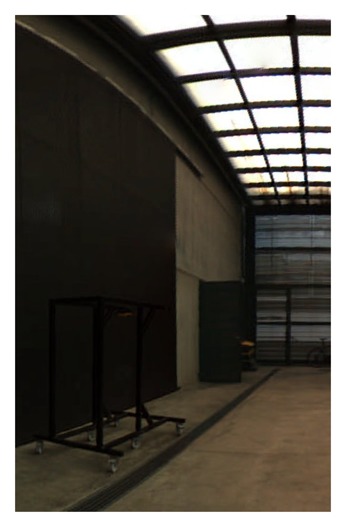
Corrected color	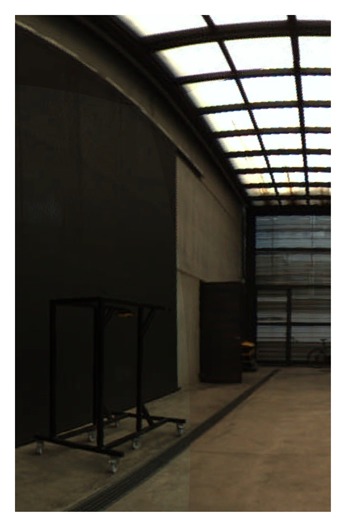	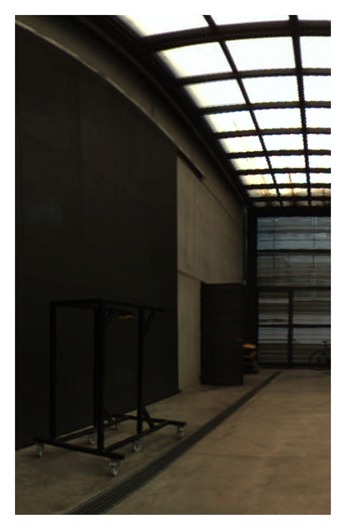	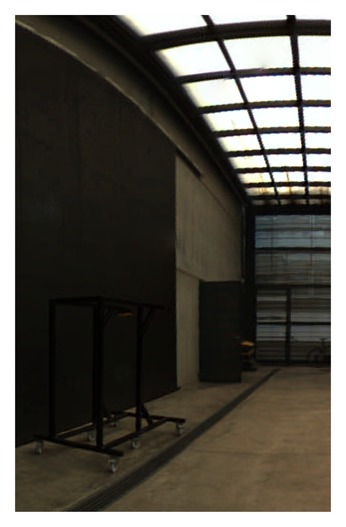

**Table 2.** Details of the same scene projected at different distances. The details are ordered by increasing distance to the camera.

		**Detail 1**	**Detail 2**	**Detail 3**
Projection Distance	*R* = 2 m	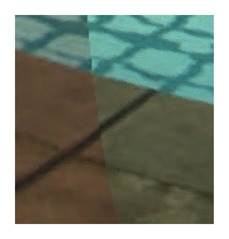	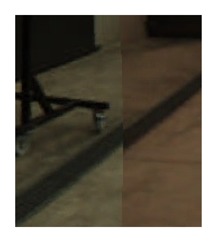	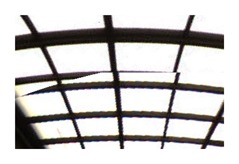
	*R* = 5 m	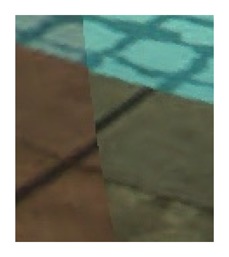	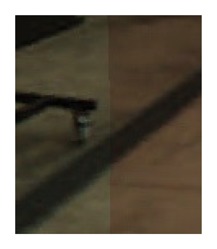	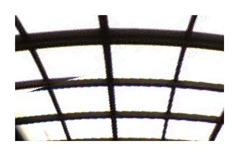
	*R* = 10 m	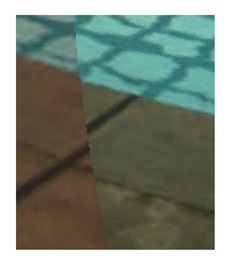	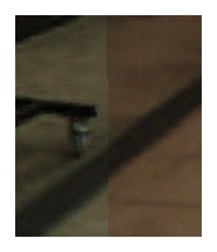	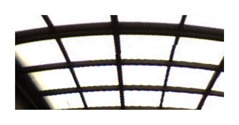

**Table 3.** Initial and refined values of the intrinsic parameter optimization for Camera 5 (2.95-mm focal length) and standard deviation of the Monte-Carlo Simulation (MCS).

**Parameter**	**Standard Calibration**	**Refinement**	**Std Deviation MCS (1000 Iterations, *σ* = 1.086 px)**
**Focal length (pixel)**	682.47	674.84	0.29
**Focal length (mm)**	3.0	2.97	0.0013
**Principal point (pixel)**	[798.66, 617.97]	[799.38, 617.9]	[0.31, 0.33]
**Distortion coefficients**	[−8.41 × 10^−4^, −1.82 × 10^−2^, 1.21 × 10^-2^, −3.7 × 10^−3^]	[−8.16 × 10^−4^, −1.1 × 10^−2^, 1.19 × 10^−2^, −5.3 × 10^−3^]	[2.69 × 10^−5^, 2.18 × 10^−4^, 2.17 × 10^−4^, 1.07 × 10^−4^]
**Number of images used**	34	11	11
**Number of checkerboard crosses/matched points**	3400	5268	N/A
**Number of features used**	3400	3494	3494

**Table 4.** Initial and refined values of the extrinsic parameter optimization and results of the Monte-Carlo Simulation (MCS). Camera 5 is selected as the global reference frame and, therefore, not included in the table.

**Parameter**	**Initial Values**	**After Optimization**	**Std Deviation MCS (1,000 iterations, *cr* = 1.33 px)**
[*α, β*, *γ*]*_c_*_(0)_ **(rad)**	[0, $\frac{\pi }{2}$, 0] = [0, 1.571, 0]	[1.26 × 10^−6^, 1.5719, 2.35 × 10^−8^]	[2 × 10^−20^, 9.22 × 10^−6^, 2 × 10^−22^]
[*α, β*, *γ*]*_c_*_(1)_ **(rad)**	[ $\frac{2\pi }{5}$, $\frac{\pi }{2}$, 0] = [1.257, 1.571, 0]	[1.2575, 1.5713, −2.57 × 10^−5^]	[5.21 × 10^−6^, 8.06 × 10^−6^, 1 × 10^−10^]
[*α, β*, *γ*]*_c_*_(2)_ **(rad)**	[ $2\frac{2\pi }{5}$, $\frac{\pi }{2}$, 0] = [2.513, 1.571, 0]	[2.524, 1.5751, 9.88 × 10^−6^]	[9.6 × 10^−6^, 9.5 × 10^−6^, 3 × 10^−21^]
[*α, β*, *γ*]*_c_*_(3)_ **(rad)**	[ $3\frac{2\pi }{5}$, $\frac{\pi }{2}$, 0] = [3.77, 1.571, 0]	[3.7705, 1.568, −4.59 × 10^−5^]	[2.2 × 10^−5^, 4.56 × 10^−6^, 1 × 10^−18^]
[*α, β*, *γ*]*_c_*_(3)_ **(rad)**	[ $4\frac{2\pi }{5}$, $\frac{\pi }{2}$, 0] = [5.027, 1.571, 0]	[5.0258, 1.5742, 1.45 × 10^−5^]	[1.42 × 10^−5^, 5.38 × 10^−6^, 1 × 10^−19^]
[*α, β*, *γ*]*_c_*_(4)_ **(rad)**	[40, 0, −50]	[39.84, −3.37 × 10^−6^, −61.83]	[8.4 × 10^−5^, 4 × 10^−20^, 5.5 × 10^−5^]
[*x*, *y*, *z*]*_c_*_(0)_ **(mm)**	[12.36, −38, −50]	[12.55, −40.27, −61.28]	[9.9 × 10^−6^, 1.2 × 10^−4^, 2.43 × 10^−3^]
[*x*, *y*, *z*]*_c_*_(1)_ **(mm)**	[−32.4, −23.5, −50]	[−32.91, −24.84, −62.81]	[7.8 × 10^−5^, 2.5 × 10^−5^, 4.9 × 10^−4^]
[*x*, *y*, *z*]*_c_*_(3)_ **(mm)**	[−32.4, 23.5, −50]	[−32.21, 22.89, −61.71]	[3 × 10^−5^, 1.5 × 10^−5^, 7.5 × 10^−4^]
[*x*, *y*, *z*]*_c_*_(4)_ **(mm)**	[12.36, 38, −50]	[13.42, 39.83, −61.39]	[2.2 × 10^−4^, 3.5 × 10^−4^, 4.9 × 10^−5^]
**Images used**		23	
**Different time frames**		9	
**Matched features**	10,564		N/A
**Features used**		3212	
**RMS error (pixel)**	8.77	1.35	N/A

**Table 5.** Initial and refined values of the housing parameter optimization and results of the Monte-Carlo Simulation (MCS).

**Parameter**	**Initial Values**	**After Optimization**	**Std Deviation MCS (500 iterations, *σ* = 3.455 px)**
**Cylinder center (mm)**	[0, 0]	[0.514, −0.679]	[0.031, 0.0438]
**Cylinder direction vector**	[0, 0, 1]	[−2.33 × 10^−3^, 6.08 × 10^−4^, 1]	[1.5 × 10^−4^, 3.4 × 10^−5^, 1.5 × 10^−4^]
**Hemisphere center (mm)**	[0, 0, 15]	[0.328, −1.47, −2.6]	[0.155, 0.1876, 0.206]
**Number of images used**		15	
**Number of different time frames**		5	
**Matched features**	7286		N/A
**Features used**		560	
**RMS of re-projection error (pixel)**	11.24	3.81	N/A
